# Intensity modulated radiotherapy: advantages, limitations and future developments

**DOI:** 10.2349/biij.2.1.e19

**Published:** 2006-01-01

**Authors:** KY Cheung

**Affiliations:** Department of Clinical Oncology, Prince of Wales Hospital, Shatin, Hong Kong SAR, China

**Keywords:** IMRT, dose optimisation, IGRT, motion compensation

## Abstract

Intensity modulated radiotherapy (IMRT) is widely used in clinical applications in developed countries, for the treatment of malignant and non-malignant diseases. This technique uses multiple radiation beams of non-uniform intensities. The beams are modulated to the required intensity maps for delivering highly conformal doses of radiation to the treatment targets, while sparing the adjacent normal tissue structures. This treatment technique has superior dosimetric advantages over 2-dimensional (2D) and conventional 3-dimensional conformal radiotherapy (3DCRT) treatments. It can potentially benefit the patient in three ways. First, by improving conformity with target dose it can reduce the probability of in-field recurrence. Second, by reducing irradiation of normal tissue it can minimise the degree of morbidity associated with treatment. Third, by facilitating escalation of dose it can improve local control. Early clinical results are promising, particularly in the treatment of nasopharyngeal carcinoma (NPC). However, as the IMRT is a sophisticated treatment involving high conformity and high precision, it has specific requirements. Therefore, tight tolerance levels for random and systematic errors, compared with conventional 2D and 3D treatments, must be applied in all treatment and pre-treatment procedures. For this reason, a large-scale routine clinical implementation of the treatment modality demands major resources and, in some cases, is impractical. This paper will provide an overview of the potential advantages of the IMRT, methods of treatment delivery, and equipment currently available for facilitating the treatment modality. It will also discuss the limitations of the equipment and the ongoing development work to improve the efficiency of the equipment and the treatment techniques and procedures.

## INTRODUCTION

For over a century, physicists and clinicians have been trying to develop ways and means of delivering doses of tumouricidal radiation, to tumours in different anatomical sites of patients. Various types of equipment and methods of treatment delivery have been developed to meet different clinical requirements. Metallic beam modifiers were first used in the 1960s to alter the spatial distribution of the intensity of the treatment beams. These have been an effective means of providing better coverage of dose to the tumours. Beam blocks, wedge filters, and beam compensators have been commonly used in 2-dimensional (2D) radiotherapy treatments. Practical means of delivering intensity modulated beams to achieve 3D dose conformity were not available until the mid 1990s. It was then that computer controlled linear accelerators with fully motorised multi-leaf collimators (MLC) were developed. In addition, 3D treatment planning computers with inverse planning algorithms for optimisation of dose were developed. Since then linear accelerator based IMRT treatment delivery systems that include the binary multi-leaf intensity-modulating collimator (MIMiC) [1], step-and-shoot MLC [2], dynamic MLC (sliding window) [3] and intensity modulated arc therapy (IMAT) [4] have been developed. They are commercially available for clinical implementation. Two other types of IMRT equipment, with different designs, namely Cyberknife [5] and helical tomotherapy [6] tool have also been developed and are commercially available.

Dosimetrically, IMRT has the ability to deliver the prescription dose to the delineated target volume with precision, while sparing the adjacent normal tissue structures. This function is like dose painting or dose sculpting [7]. However, such a degree of precision and conformity with dose may not be realised clinically. This is because of uncertainties in delineating and contouring the target and normal tissue structures, treatment set up errors, patient and organ movements, geometrical tolerance of the treatment machine, and dosimetry calculation errors. The purpose of this paper is to review the dosimetry advantages of the IMRT, clinical benefits that have been achieved so far, issues related to clinical implementation of the technique, and limitations of current equipment and clinical procedures in large scale implementation of the modality as a standard treatment. This paper will also discuss the research and development work being conducted to resolve some of these problems.

## ADVANTAGES OF IMRT

**Figure 1:**
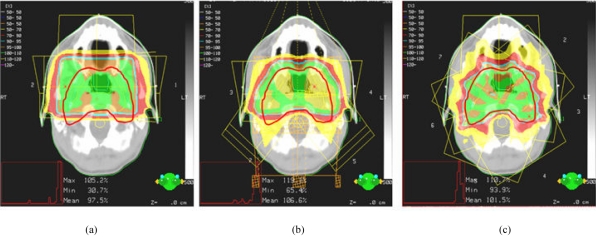
A dosimetry comparison between (a) a 3-beam conventional 2D treatment, (b) a 6-beam conventional 3D conformal RT treatment, and (c) a 7-beam IMRT treatment. The PTV is represented by the solid red line. The 100% and 70% of the prescription dose are shown by the green and red colour-washed areas. A better dose conformity to the PTV can be achieved in the IMRT treatment.

IMRT has attracted wide spread interest because of its dosimetric and potential clinical advantages (Figure 1). Numerous dosimetry studies on linear accelerator based IMRT treatments of different anatomical sites have been reported, and all of them show that IMRT can have definite dosimetry advantages over 2D and conventional 3DCRT treatments [8-18]. Whether the dosimetric advantages of IMRT can be realised clinically would depend on a number of factors, including (a) the accuracy in localisation and delineation of the tumour and the adjacent critical tissue structures, (b) understanding of the optimum relationship between dose and response for the individual tumour, and (c) delivery of the prescription doses according to the treatment plans. These are challenging requirements that need to be met. Some of the research and development work aiming to address these and related issues are discussed below.

IMRT's high conformity with dose facilitates escalation of dose and better protection of normal tissue structures. These features make it particularly suitable for the treatment of diseases that involve high rates of local recurrence and toxicity and complications related to treatment. Nasopharyngeal carcinoma (NPC) is a cancer disease that can benefit from the treatment because of the recognised radio-curability and the evidence of a relationship between dose and response for the disease. The numerous critical normal tissue structures in close proximity of the tumour also warrant this treatment [8, 19-25]. It is difficult to deliver a satisfactory radiation dose distribution to the NPC target volume by conventional radiotherapy techniques without significantly irradiating the critical tissue structures. This is particularly difficult in locally advanced disease [26-27]. Planners of treatment often have to make compromises between protection of normal organ and optimal coverage of dose. IMRT technique has been implemented routinely in our clinic since July 2000, with the aim of improving the dosimetry problem in NPC treatment. Over 300 patients with early or advanced stages of NPC have been treated by means of the DMLC IMRT technique [8, 24]. Our early treatment outcome is encouraging and confirms the promising role of IMRT [24]. A 3-year local control rate of 92% and overall survival of 90% were achieved with a standard dose of 66 Gy to the gross tumour volume (GTV), with limited acute and late toxicities. It is expected that further improvement can be achieved with escalation of dose using the IMRT. Escalation of dose by simultaneous integrated IMRT boost to a tumour dose of 76 Gy for treatment of locally advanced NPC has been reported by another centre with good short term outcome [25]. The 2-year local control and overall survival reported are 96% and 92 %, respectively. Excellent short-term results have also been achieved by other centres using IMRT for treatment of NPC [28-29], with high rates of local control of 97% and overall survival of 88% to 97%. Furthermore, the early clinical data indicate that the treatment can better spare the parotid gland, compared with conventional treatments [22,24,30]. Encouraging clinical results have also been reported on using IMRT for the treatment of a number of other tumour sites, including prostate, breast, oropharyngeal, vulvar, and anal [31-39]. While survival data are still pending, the main clinical advantages reported are reduction of damages to normal tissue structure caused by treatment.

## METHODS OF DELIVERING IMRT TREATMENT

**Figure 2:**
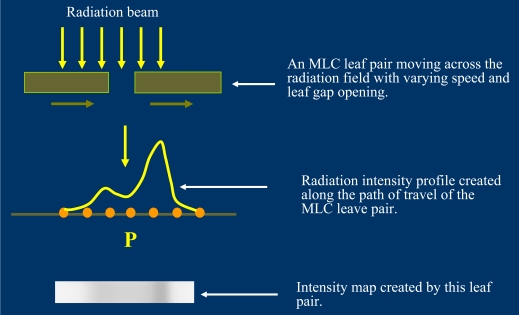
Fluence intensity map created by a pair of MLC leaf pair sliding across the radiation field.

**Figure 3:**
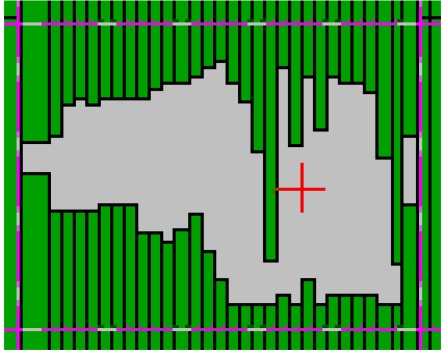
Typical pattern of movement of the MLC leaf pairs when operating in the dynamic MLC mode (available for download from http://www.biij.org/2006/1/e19).

IMRT treatments are primarily delivered by linear accelerators (linacs) with multi-leaf collimator (MLC) systems. The equipment can be commissioned to deliver IMRT treatment in different operation modes using MLC. One of the most commonly used modes of operation is the step-and-shoot or segmental MLC (SMLC) technique [2], in which, the modulation of intensity of beam in a treatment field is created by the exposure of a series of MLC shaped discrete segmental fields. The radiation beam is turned off when the MLC leaves are moving from one field segment to another and is turned on only when the leaves reach and stop at the designated segment positions. The method is similar to two-dimensional dose painting by the individual segmental fields to create a composite IMRT beam of the required pattern of intensity. The other commonly used mode of IMRT delivery is the sliding window or dynamic MLC (DMLC) technique [3]. The DMLC IMRT beam is created by moving the individual leaf pairs of the MLC system across the treatment field when the radiation beam is turned on. The required pattern of intensity fluence for the IMRT beam can be achieved by varying the width of the gap between each of the leaf pairs and the speed of travel of individual leaf pairs (Figure 2). Figure 3 shows a video clip of a typical pattern of movement of the MLC leaf pairs when operating in the dynamic MLC mode.

The Peacock MIMiC serial tomotherapy system developed by NOMOS Corporation is also a widely used IMRT delivery system [1]. The MIMiC multileaf system, which is a slit type collimator, is mounted onto the treatment head of a linac and replaces the linac collimator system when in operation. Dose delivery is made through a narrow slice of the patient using arc rotation. Beamlets of varying intensity can be created by switching the individual leaves of the MIMiC multileaf system in and out on a binary basis. This is done when the radiation beam is turned on and the gantry rotates are around the patient. Modulation of intensity of radiation beam is achieved by varying the opening time of the individual leaves during gantry rotation. On completion of a gantry rotation the radiation beam is switched off, and the patient is shifted longitudinally by moving the couch to treat the next adjacent axial slice. The process is repeated until treatment of the whole target is completed.

The helical tomotherapy, which was developed by Mackie *et al*. [6] at the University of Wisconsin, has gained popularity. The treatment unit has a mega-voltage linear accelerator waveguide mounted onto a computed tomography (CT) gantry. The gantry and couch motions of the machine are similar to that of a single-slice spiral CT. A binary MLC unit similar to that of the NOMOS MIMiC is used for collimation of beam and modulation of intensity during treatment. Modulation of intensity of radiation beam is achieved by varying the leaf opening time and gantry speed and moving the treatment couch like a helical CT. A set of CT detector rows is installed as in a conventional CT, to provide on-line mega-voltage CT imaging.

A robotic linac, the Cyberknife [5], which was developed by Accuray Inc. (Sunnyvale, CA, USA), is a linear accelerator based high precision stereotactic radiotherapy treatment machine. It consists of a miniature linear accelerator that operates at a frequency about three times higher than those of conventional linear accelerator machines. The miniature accelerator is mounted on an industrial robotic arm to provide a highly flexible 3D frameless stereotactic radiosurgery delivery system. It also has a pair of orthogonal on-line fluoroscopy x-ray imaging systems that localise the treatment target in a coordinate system. The spatial information is then fed back to the robotic arm to direct the radiation beams stereotactically to the target volume located at the isocentre. This treatment can be considered as IMRT because a large number of small pencil beams of different intensities can be directed to the target volume from different angles, to deliver the required distribution of dose. The treatments are delivered stereotactically, with feedback of any organ motion to the robotic arm.

**Figure 4:**
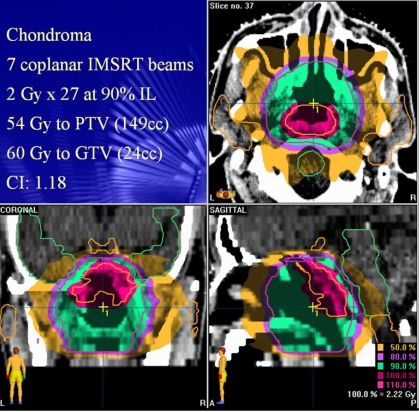
The dose distribution of an IMSRT treatment of a chondroma. A higher dose (shown in red colour-washed area) can simultaneously be delivered to the main bulk of the lesion while the rest of the PTV is given the normal dose (shown in green colour-washed area). This is a simple form of dose painting or sculpting.

**Figure 5:**
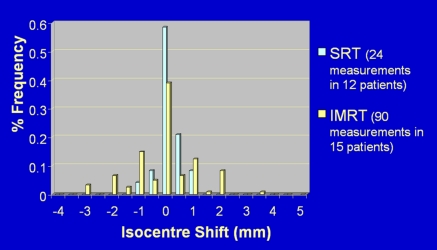
Inter-fraction treatment set up errors (shift in isocentre) in the lateral direction of IMRT treatments (represented by the yellow histogram) and stereotactic treatments (represented by the blue histogram). Similar results are find in the superior-inferior and the anteria-posteria directions. The data confirm that stereotactic set up can reduce the amount of inter-fractional geometrical errors.

Linac based intensity modulated stereotactic radiosurgery (IMSRS) or radiotherapy (IMSRT) techniques using small leaf MLC of less than 5 mm leaf width have been developed to improve the conformity of conventional stereotactic treatments with dose. This technique utilises the high stability and high precision patient immobilisation and target localisation systems of conventional SRT/SRS and the finer resolution of small leaf MLC system to further improve the conformity of the treatment with dose, compared with conventional IMRT (Figures 4 and 5). This technique can better protect the critical tissue structures that are in close proximity to the treatment target. Therefore, brain, head, neck, and spinal cancers can be treated by utilising this technique [40-41].

## TREATMENT PLANNING AND SIMULATION

**Figure 6:**
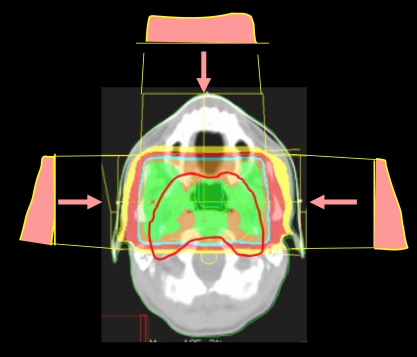
Principle of conventional forward planning. The planner starts with a set of beam weights and profiles to obtain a plan by trial-and-error process.

**Figure 7:**
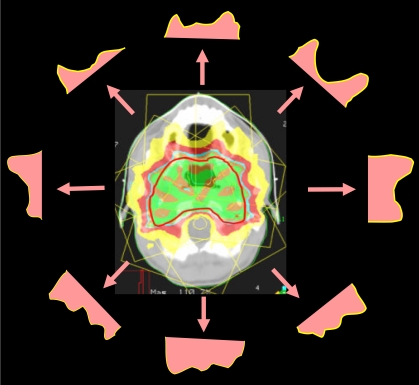
Principle of inverse planning- The planner define the required dose & dose distribution for treatment and the computer can calculate and optimised the beam intensity patterns of the individual IMRT beams to meet the dose requirements.

The work by a number of authors [42-48] on inverse planning of treatment and optimisation of dose is pivotal to the development and the implementation of IMRT. In conventional forward treatment planning, the planner selects by experience the required number of open or wedged treatment beams of appropriate beam geometries. The TPS calculates the composite distribution of dose by adding the dose contributed by each of the treatment beams. If the dose and the distribution of dose are unsatisfactory, the planner varies the beam parameters and geometries and repeats the calculation. The processes are repeated until an acceptable treatment plan is achieved. In inverse planning, the planner specifies the required dose and the distribution of dose for the target volumes and the acceptable tolerance dose for individual normal organs of interest, in the form of a constraint table for dose or template for the TPS. This is done to calculate the pattern of beam intensity or fluence map of the individual treatment beams that are required to achieve the specified dose and the distribution of dose (Figure 6 and 7). The treatment planner needs to specify for each IMRT beam the required MLC opening that covers the target volume, the gantry and collimator angles, in addition to the dose specification. Upon satisfactory calculation of the required map of beam intensity, the TPS can generate for each of the beams, a set of MLC leaf motion sequence codes. These can be transferred to the linac MLC controller to drive the individual MLC leaf movements to achieve the required map of beam intensity and, therefore, the dose and the distribution of dose during treatment. The degree of sophistication of the treatment plan depends on the number of critical normal organs requiring protection, the shapes of these organs, the treatment target, and the geometrical margins available between the normal organs and treatment target. The inverse planning system may not always be able to generate a satisfactory treatment plan based on a given constraint table for dose. The planner of treatment may need to change the constraint parameters of dose and repeat the iteration process for optimisation of dose several times before a satisfactory plan can be achieved. To reduce the number of the optimisation process and, therefore, minimise planning time, a universal or optimised constraint template of dose, for individual target sites, is required. This is very difficult to achieve in practice for complicated treatment sites, such as, NPC in which a large number of critical tissue structures are required to be protected. A TPS which can optimise the constraint parameters of dose during the optimisation process of dose needs to be developed. Another important development in planning technology for treatment, which helps the implementation of IMRT, is the availability of several tools for evaluation of plans. These tools can be used for quantitative assessment and comparison of treatment plans. Tools for evaluation of plans, such as, dose-volume-histogram (DVH) and dose conformity index (CI), in addition to 3D dose and distribution of dose analysis tools, are available in most planning systems for plan evaluation. Software tools based on mathematical models of tumour control probability (TCP) and normal tissue complication probability (NTCP) are available to calculate these biological indices from DVH data. Such information can serve as useful reference for planners of treatment, in optimisation of dose and evaluation of plan.

The availability of CT simulator [49], MR simulator [50], and PET-CT simulator [51] facilitates accurate 3D localisation and delineation of target, virtual treatment simulation, and verification of radiotherapy treatments for different target sites. Therefore, the geometric and the dosimetric accuracy of radiotherapy treatments improve further.

## ISSUES IN CLINICAL IMPLEMENTATION OF IMRT

### High cost

Clinical implementation of IMRT requires the availability of a range of sophisticated and high cost equipment, as well as a range of compatible supporting facilities, such as, imaging equipment, computer networks, dosimetry and quality assurance (QA) systems, immobilisation system for patients, and a multi-disciplinary team of well trained staff. These are expensive to establish.

### Complex and time consuming procedures

Proper verification of dosimetry and treatment QA procedures are important measures to ensure the treatment can be delivered according to the treatment plan. This is one of the limiting factors for large scale implementation of IMRT because of the amount of work involved, physics work in particular. Early IMRT techniques for verifying dosimetry were based on measurements of dosimetry for individual patients. A typical procedure for verification of treatment plan was to transfer the treatment plan to a specially designed measurement phantom [54], by replacing the patient with the phantom at the TPS. Ionisation chamber and film and/or TLD measurements were then performed with the phantom irradiated according to the treatment plan. The measured dose and the distribution of dose were then compared with that of the TPS calculated phantom plan to verify the integrity of the treatment. This type of method for verifying dosimetry usually involved tedious and time consuming measurements of dosimetry [55-57]. During NPC treatment at our centre, a physicist usually took about eight hours to do a full verification of dosimetry on an IMRT plan, with about three machine-hours for measurement of dosimetry. This used to be one of the bottlenecks in the workflow of our IMRT programme. Therefore, the concept of virtual verification was developed in which the monitor unit and the fluence map of each of the IMRT beams as calculated by the TPS, were verified by means of an independent MU calculator and beam fluence generator [58-59]. This concept works only if the treatment machines, the MLC leaves in particular, can operate properly and the MLC leaf sequence files can be transferred from the inverse planning system to the treatment machines, correctly. This demands that stringent QA tests be implemented to test the functionality of the MLC system and the integrity of the network system. The idea is to replace as much as practicable, QA procedures that are patient specific with procedures that are equipment specific. Although full or partial verification of dosimetry may still be required to be performed on some of the plans on a randomly sampled basis, virtual dosimetry verification can reduce the patient specific QA time to a more manageable amount. Similar types of MU calculators are now commercially available.

Verification of treatment field portal is another important QA procedure in IMRT. The procedure is disease specific depending on, for example, the amount of the inter-fraction and/or intra-fraction target movement, although the objectives are the same. For static treatment targets, the QA measures are mainly concerned with the verification of the treatment field portals by, for example, comparing the portal film images or the field portal taken by the electronic portal imager (EPI) with the reference field portals which usually consist of the digital reconstructed radiographs (DDRs) created at the TPS or CT-simulator or the conventional treatment simulator images. EPIs can also be used as dosimetry detectors for on-line electronic portal dosimetry (EPD) system [61-62]. This will enable on-line verification of delivery of dose in IMRT treatment [63-64]. Linear accelerators with built-in EPD systems are now commercially available. These may help to improve the efficiency and the accuracy of verification of dosimetry and treatment QA procedures. For mobile targets, the procedures would be more sophisticated and additional measures are required to ensure accurate localisation of the target volume. Some of the developments in correction of inter-fraction and intra-fraction target movements are discussed below.

### Patient immobilisation and target localisation

**Figure 8:**
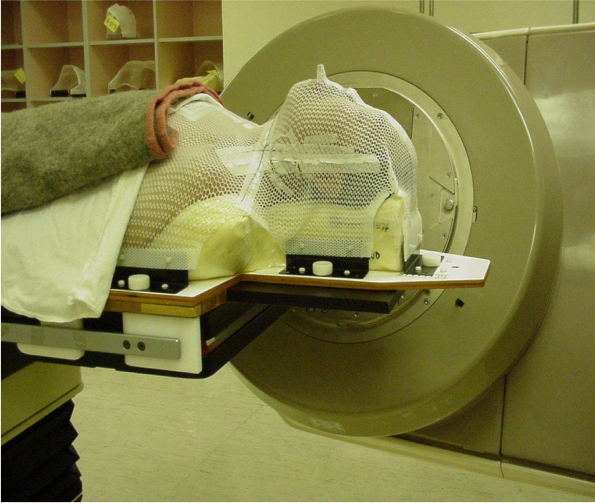
A thermal plaster patient immobilisation cast used in IMRT treatment.

**Figure 9:**
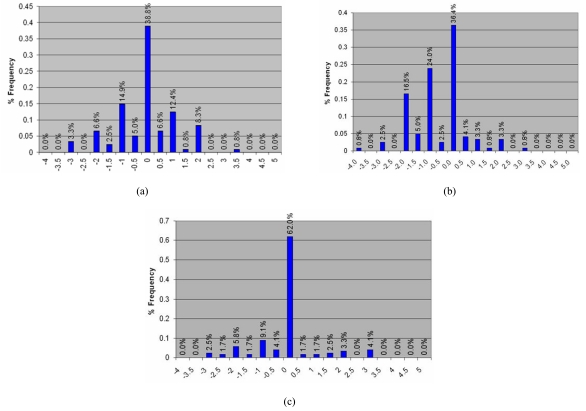
Stability of thermal plaster cast immobilisation system for NPC treatment. The diagrams show the frequency distribution of inter-fraction treatment positioning errors due to isocentre shift in the (a) lateral direction, (b) anterior-posterior direction and (c) superior-inferior direction. Frequency distribution of for patient immobilisation in IMRT treatment.

IMRT treatments are more sensitive to geometrical errors, compared with conventional 2D and 3D treatments because of their higher dose conformity indices. The stability and the precision of the patient immobilisation system need to be considered in determining the amount of treatment margin required for proper coverage of target and adequate protection of normal critical tissue structure. These factors about the system need to be maintained throughout the course of the treatment. A well designed and carefully prepared thermal plaster immobilisation cast should be comfortable for the patient and should be able to achieve an inter-fraction and intra-fraction patient positioning accuracy of within 3 mm throughout the course of the fractionated IMRT treatment (Figures 8 and 9).

### Dedicated QA programmes for equipment

The performance of the machines used for treatment and the accuracy and the stability of their dosimetry and MLC systems in delivering the intensity modulated beams, are critical in IMRT treatment. This is particularly important in the implementation of the virtual dosimetry verification system, which assumes the proper and accurate operation of the machines used for treatment. Dedicated and stringent QA programmes have been developed for equipment and reported by various authors. These include the design to check and ensure the proper operation of the machines used for treatment in IMRT beam delivery, especially for checking the performance and the integrity of the MLC system [54-65] operating in the dynamic IMRT mode.

### The need for accurate target delineation

**Figure 10:**
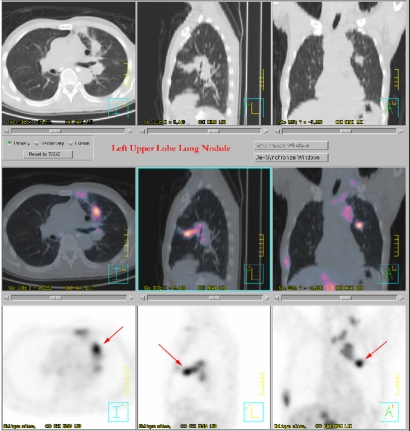
A PET-CT image can provide more accurate diagnostic and staging information on a lung tumour for IMRT treatment planning (courtesy of Dr. Hector Ma, St. Teresa's Hospital, Hong Kong)

**Figure 11:**
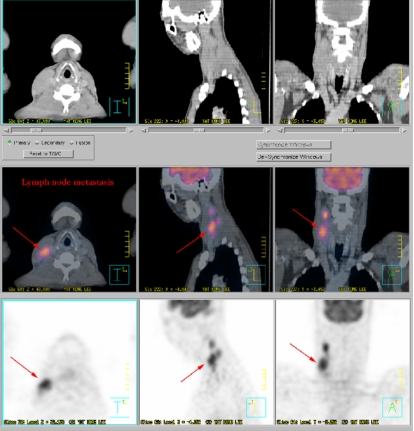
The fusion of CT and PET provide more accurate information for IMRT treatment planning. In this example, the spread of lymph mode metastasis of a nasopharyngeal carcinoma is can be clearly identified (courtesy of Dr. Hector Ma, St. Teresa's Hospital, Hong Kong)

Accurate determination of the target volume and the geometry of the organs at risk (OAR) is another essential requirement in IMRT. The dosimetry advantages of IMRT treatment may be realised clinically only if anatomical information on the geometries and locations of the target volume and organs at risk (OAR) are delineated with the required precision. This information is essential for planning treatment and calculating dose, as well as for guiding the delivery of treatment. CT images have the advantage of high spatial integrity and good spatial resolution. In addition, they provide information on electron density required for calculating dose of radiation. Fairly accurate delineation of the target and contouring of the OAR can be achieved with CT images in most situations. In some situations, CT images alone cannot accurately define the entire extent of the tumours [66]. Progress in MRI and PET imaging technologies and in image registration techniques, such as, multimodality image fusion, has facilitated the accurate determination of the extent of gross tumour and the critical tissue structures of interest. CT, in the past two decades, has played an important role in the planning of radiotherapy treatment. MR images, because of their superior contrast resolution for soft tissues, were widely used for delineation of tumour in the past decade. The current availability of software tools for fusing MR and CT images can further improve the accuracy of contouring and delineation of soft tissue structures [67-68]. CT and MRI fused images have been found to improve the determination of gross tumour volume (GTV), clinical target volume (CTV), planning target volume (PTV), and organs at risk (OAR) in NPC planning [69]. Functional MRI is another useful tool that can provide information on the activities and functional map of the brain, which in turn allows better delineation of the brain tumours and the sensitive functional regions of the brain [70]. The inadequacy of CT in delineation of tumour volume can in most cases be partially overcome by MR imaging. Positron emission tomography (PET) is another imaging modality that can enhance the accuracy of localisation of target and contouring for planning IMRT treatment. Studies by Grosu *et al*. [71] in patients with brain tumours have shown that, compared to CT and MRI alone, the image fusion of CT or MRI and amino acid SPECT or PET enables a more correct delineation of GTV and PTV. The use of F-18 labeled fluorodeoxyglucose positron emission tomography (FDG-PET) imaging has been found to improve significantly the diagnosis and the staging of cancers, such as, lung cancers, compared with CT alone [72-73] that helps to improve accuracy in delineation of target volume (Figure 10). The availability of integrated PET-CT can further improve the accuracy of diagnosis and staging of cancer disease [74]. A potential benefit of PET-CT based planning is its ability to exclude or include CT suspicious lymph nodes from the target volume [51] (Figure 11). While FDG-PET has been found useful in defining the nodal extension for planning lung treatment, the usefulness of the current equipment in improving the accuracy of delineation of highly inhomogeneous moving target, such as, lung tumour, is still to be investigated. This is because of issues, such as, uncertainties in defining the tumour edge in PET scans, limitation in spatial resolution, and motion of tumour [75].

### Unresolved problems in organ contouring

**Figure 12:**
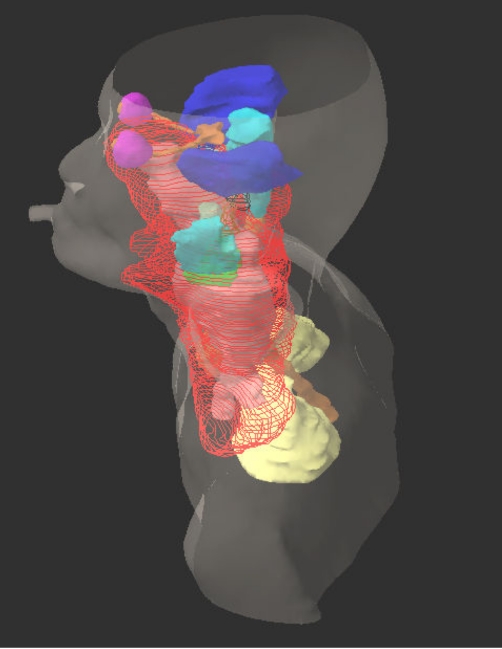
Some of the critical normal organs of interest in NPC treatment. The PTV is contoured in red.

Contouring of targets of treatment and OARs is a tedious and time consuming process in IMRT because of the large number of CT images involved and the level of precision required. The problem is more serious in head and neck cancers, such as, NPC that requires the contouring of more then 30 structures on as much as 100 CT slices, typically, 2.5 mm thick (Figure 12). It usually takes an experienced radiation oncologist about one hour to contour the targets of treatment and a further one to two hours to contour all the relevant critical normal tissue structures. The current generation of automatic segmentation software tools is not very helpful in contouring some of the critical normal soft tissue structures that do not have sufficient CT number differentiations at the boundaries. One option to reduce the contouring time is to use less CT images by using thicker CT slices of 5 mm instead of 2.5 mm. This, however, can introduce significant dosimetry errors in some of the serial organs, such as, brain stem and optic nerves in NPC treatment plans, especially in locally advanced disease. Until more efficient and accurate segmentation software tools are available, delineation of target and organ remains to be one of the limiting factors in large scale implementation of IMRT.

### Management of interfraction target movements

The day-to-day positional changes in the target volumes of some cancers such as prostate, cervix, bladder, and rectum, can be a limiting factor in IMRT treatment. This is particularly true for escalation of dose, which is more sensitive to uncertainty in treatment than conventional 2D and 3DCRT treatments [76-77]. Interfraction organ motion of this sort, which can cause significant dosimetric deficiencies in the target volumes, is commonly accounted for by using appropriate margins when contouring the PTV [78]. For highly conformal treatments, such as, IMRT, the required margins can be relatively quite large [78-79], and is a trade off for the type of treatment. Correction techniques using in-room ultrasound or CT-guided adjustment of positions for treatment before delivery of treatment have been developed to minimise the effects of interfraction organ movements [77,80]. Adaptive treatment techniques have also been developed aiming to account for interfraction organ movement in high precision radiotherapy [81-82]. In this technique, a continuous adaptation of the treatment plan was made, based on anatomical information obtained through daily CT images of the movement of the PTV over time. The technique aims to optimise the coverage of target and minimise the amount of irradiation of normal tissue. Adaptive technique using an in-room integrated CT-linear accelerator has also been developed [83].

**Figure 13:**
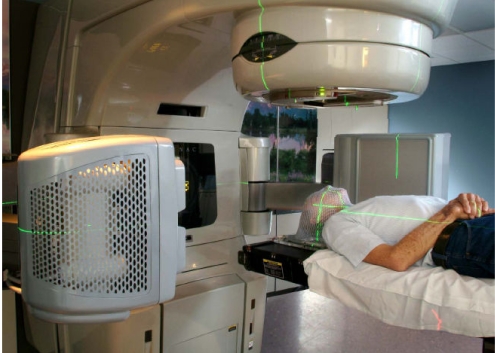
A linear accelerator with built-in kV cone beam CT system for IGRT treatment delivery (courtesy of Varian Medical Systems)

**Figure 14:**
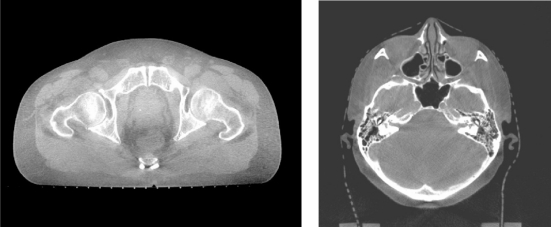
CT images produced by the cone beam CT system of a linear accelerator (courtesy of Professor Lei Xing, Stanford University School of Medicine, USA)

**Figure 15:**
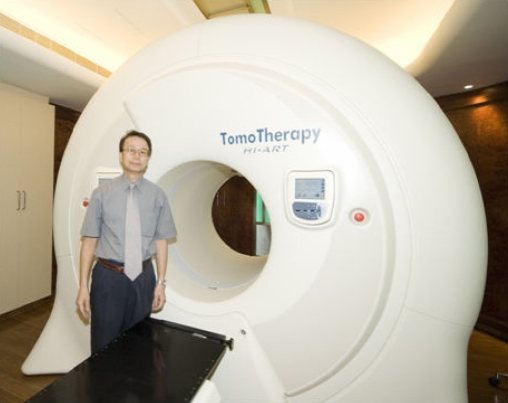
A tomotherapy unit (courtesy of Hong Kong Sanatorium & Hospital, Hong Kong)

**Figure 16:**
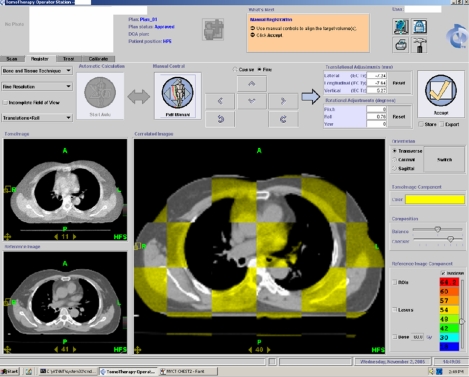
On-line treatment verification by matching of the planning CT image (bottom left) with the tomotherapy treatment set up image (top left) immediately before treatment delivery without moving the patient (courtesy of Hong Kong Sanatorium & Hospital)

Inter-fraction target position changes and set up errors can be minimised by using the image-guided radiotherapy (IGRT) technique. Linear accelerator based IGRT system with add-on or integrated cone beam CT imaging facilities with x-ray operating at kV or MV energy have been or are being developed for verification and correction of beam geometry. The cone beam CT system can produce high quality CT images that can enable target matching and correction of position immediately before treatment (Figures 13 and 14). The Helical tomotherapy system (TomoTherapy Inc., WI, USA) provides helical CT image guided IMRT treatment without changing the patient's position throughout the treatment and the imaging processes. The mega-voltage CT imaging system can produce good quality CT images for verification of target position prior to treatment (Figures 15 and 16).

### Management of intrafraction target movements

**Figure 17:**
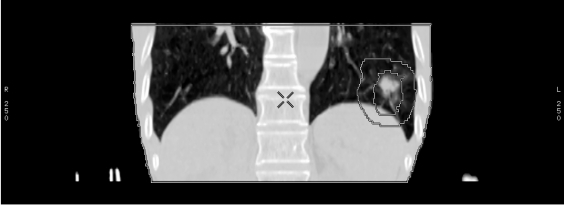
Video clip on a lung tumour movement during a respiratory cycle (available for download from http://www.biij.org/2006/1/e19).

**Figure 18:**
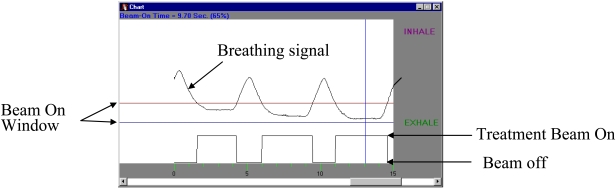
Gating of radiotherapy treatment beams by respiratory motion waveform to compensate for target movement.

One of the major challenges in treating tumours in the cardio-thoracic region, as in the case of lung, pancreas, or liver cancers, is the respiratory induced movement of target during treatment. Figure 17 shows a video clip of lung tumour movements in one respiratory cycle. The respiratory motion of the lung can displace the target tumour away from the treatment field portal, resulting in inadequate coverage of dose for the tumour. To ensure satisfactory coverage of dose for the target volume, a large margin in excess of 2 cm was required to be added to the clinical target volume (CTV) in lung treatment [84-85]. This resulted in a larger volume of normal lung tissues to be irradiated, which in turn, increased the probability of morbidity and limit the dose that can be safely given. Such intra-fractional movement of target is often a limiting factor for dose in IMRT treatment and, particularly, escalation of dose. The current generation of cone beam and helical CT imaging systems cannot be used readily for correcting geometrical errors due to movement of patient and motion of organ during treatment. Specially designed respiratory control equipment that can be used to limit or compensate for motions of organ, is now commercially available. One such equipment is the stereotactic body frame developed by Elekta AB (Sweden) [86]. The system restrains the breathing volume of the patient and, therefore, limits the movement of the target during treatment. The active breathing control (ABC) system developed by Wong *et al*. [87] is another system that can be used to control and hold the patient's breathing so as to immobilise the target of treatment for irradiation. Another motion compensation system, which is currently in clinical use in our hospital, is the RPM respiratory gating system developed by Varian Medical Systems, Inc. The system operates in conjunction with a diagnostic 4D CT scanner that is used to acquire a set of respiratory gated CT images, and at the same time the corresponding waveform motion of an infrared marker on the chest surface, as detected by a camera (RPM waveform), is recorded. The organ's motion as shown by the 4D CT images is then correlated with the RPM waveform that can be used for compensation of motion during treatment (Figure 18). These two types of compensation systems for respiratory motion have several limitations. They are unsuitable for treating patients who are not cooperative and patients who have unsatisfactory physical conditions. The exact location of the target of treatment and the dynamics of its motion on the day of treatment cannot be verified accurately unless a CT scanner is available in the treatment room.

**Figure 19:**
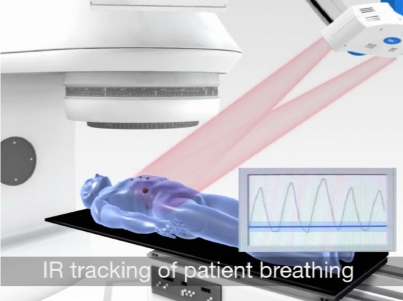
Video clip on principle of the BrainLab Exac-Tract X-ray treatment (courtesy of BrainLab, Germany) (available for download from http://www.biij.org/2006/1/e19).

Another compensation method for motion of organ is the IGRT technique. It is based on a bi-plane x-ray fluoroscopy target localisation and tracking system, such as, the Exac-Trac X-ray (BrainLAB AG, Germany) imaging system (Figure 19). As shown in the video clip, the orthogonal bi-plane x-ray fluoroscopy imaging system localises the target and tracks its movement during treatment. The radiotherapy treatment beam is turned-on when the target enters the range of field coverage of the treatment beam and is turned-off when the target is outside the field coverage. The x-ray target tracking system can localise the target volume accurately and provide information on the dynamics of its movement. This can be used to guide the treatment.

### Radiation safety

As compared with conventional treatments IMRT treatments, in general, require the use of more machine monitor units (MU) per target dose. In the case of NPC treatments using the sliding window technique, a factor of 5 or more MU is used in IMRT, compared with conventional 2D or 3D treatments. This, in turn, will cause a higher integral dose to be delivered to the normal tissues of the patient, in addition to the fact that IMRT generally used more radiation beams. This can result in an increased risk of malignancies induced by secondary radiation [88-89]. The radiosensitivity of the normal tissues can also be enhanced, increasing normal cell-kill. This, in turn, can contribute to late toxicity and reduced therapeutic ratio [90]. As a much larger number of MUs are used on each patient undergoing IMRT, the adequacy of the room shielding should also be assessed for radiation safety prior to implementation of the treatment modality. The corresponding increase in head leakage of treatment machine and scatter radiation must be taken into consideration for secondary shielding, which would depend on the patient load in IMRT [91]. For the same reason, IMRT treatments using high energy photon beams, above 10 MV, can have more serious problems of neutron activation. The ambient background radiation in the treatment room can be significantly elevated following the treatments and thus causing higher staff dose.

## FUTURE DEVELOPMENTS AND APPLICATIONS

### Image registration and segmentation tools

A major bottle-neck in the IMRT work flow in a busy centre is the registration of image and contouring of organ. The current generation of image registration and contouring software is slow and tedious. Better segmentation tools are needed for large scale implementation of IMRT in such centres.

### Biological imaging guided radiotherapy

IMRT has the capability to paint or sculpt the dose of radiation to conform to the geometries of different sub-targets within a PTV. The current limitation is in the ability of the planners to delineate such sub-targets accurately. The advancement in imaging technology and technique can help to characterise the tumours and delineate the volumes of iso-sensitivity to dose. Functional imaging techniques, such as, MR spectroscopy, SPECT, and PET can provide metabolic and functional imaging of hypoxia, cell proliferation, apoptosis, tumour angiogenesis, and gene expression. This can enable identification of differently aggressive areas of a biologically inhomogeneous tumour mass that can be individually targeted using IMRT. Therefore, a biological, inhomogeneous distribution of dose can be generated, the so-called dose painting or dose sculpting within the PTV, so as to improve the therapeutic ratio of the treatment [7, 71]. Feasibility study of this technique should be carried out.

### IGRT for compensation of motion or tracking of target

Various methods of IGRT are being developed to manage or compensate for movements of organ and errors in the positioning of patient. Progress has been made in correcting inter-fraction positional changes of organ between treatment sessions. A good solution to compensate for intra-fraction movements of target, without having a long treatment delivering time has yet to be developed. The current methods for tracking of target and gated radiotherapy are complex and inefficient. While they may be applicable for guiding 3DCRT treatments, they are impractical for implementation in IMRT treatment. Further research is required before the technique can be applied in IMRT.

### Automation in treatment and dosimetry QA procedures

Automation in positioning of patient and in set up based on internal and external marker tracking is available for accurate and efficient delivery of treatment. EPI with solid state flat panel detectors are becoming a matured technology for routing implementation of on-line verification of field geometry. Although on-line electronic port dosimetry system is becoming commercially available, practical, efficient, and reliable automated on-line treatment dosimetry QA is yet to be developed. This technology can help to simplify some of the QA procedures and facilitate large scale clinical implementation of IMRT and other high precision treatments.

### Clinical applications and studies

There is, in general, a lack of published data on the results of randomised clinical studies to prove the efficacy of IMRT. This is partly because the treatment modality is still relatively young, and meaningful long term clinical follow up data have yet to be collected and analysed. Another possible reason for this may be the lack of drive in the radiotherapy community to conduct randomised trials. The lack of drive could be because of the obvious dosimetric advantages of IMRT over conventional treatments and the encouraging early clinical results of the treatment. However, it is expected that clinical results of randomised trials will be available in the coming years, including those of NPC trials that are being conducted in Hong Kong.

It has been shown in a recent retrospective study on the pattern of local failure in a group on non-metastatic NPC patients [95] that improvement in target localisation or dose conformity alone, without dose escalation, can only avoid less than 20% of the local failure that is attributable to radiographic miss or sub-optimal target coverage. Within-field failure was found to be the predominant mode of local failure, which indicated that there was a relationship between dose and response in NPC patients. In addition, the strategy to escalate dose, used to increase the physical dose to the tumour bed in NPC of advanced T-stages appears to have clinical benefits [22,24-25]. These observations may form the basis for randomised studies to be carried out to address the issue of optimal dose in NPC treatment using IMRT. Optimisation of dose distribution within the PTV, taking into consideration characteristics of the tumour cells in different parts of the PTV (e.g. tumour burden, proliferation, and hypoxia) in NPC treatment, should be investigated.

Emerging data have indicated that there is a dose-response for non-small cell lung carcinoma. [92-94]. It may be a potential treatment site for dose escalated treatment using IMRT. The maturing technology of 4DCT, respiratory gated target motion compensation, and immobilisation of target by breathing control during treatment, facilitate safe delivery of a highly conformal escalated dose of radiation to the target. Improvement in planning computer dosimetry algorithm can further improve the accuracy and conformity of delivering dose to the target, in the lung. Research and development work on image guided on-line real time target tracking treatment compensation systems is being conducted. If this materialises, the problem of intra-fraction motion of organ can be resolved and the therapeutic ratio of lung treatment can be improved.

## CONCLUSION

IMRT has shown to have dosimetry advantages over conventional 2D and 3DCRT treatment techniques in a number of cancer sites. Clinical data are beginning to show that the treatment is safe and effective. It appears that IMRT is more beneficial for: a) disease sites that have recognised radiocurability and evidence of a relationship between dose and response for the escalation of dose; and b) the numerous critical normal tissue structures in close proximity to the tumour that preclude the use of other treatment techniques. The technique has shown to have survival and other clinical benefits in treatment of NPC, compared with conventional 2D treatments, and a large scale implementation of the technique for treatment of this disease appears to be fully justifiable. While survival benefit remains to be seen, the technique has benefited treatment of prostate cancer by reducing complications related to treatment, compared with conventional 2D treatment. This suggests that replacing 2D treatment with IMRT can be justified. Clinical data reported on a number of other disease sites also demonstrated similar benefits in the reduction of complications related to treatment It is expected that more sites of treatment will be found to have benefited from the treatment when more clinical data are available. However, due to limitations of current equipment, it is not expected that all the dosimetry benefits of IMRT will be fully realised clinically in the near future. Perhaps, not until practical solutions for accurate and correct delineation of the target volumes, proper compensation for motions of organ during treatment, and change in position of these structures with time can be found and corrective measures be clinically implemented. The research and development work being conducted by academic institutions and manufacturing industries and the exciting progress being made in the areas of biological imaging, dosimetry techniques, and image guided IMRT look promising for improving the efficiency and effectiveness of the technique. The modality of treatment is expected to have a positive impact on the clinical outcome, especially in locally advanced cancer diseases. This impact will be in terms of reduction of complications related to treatment and increase in overall survival rates when these technology become more matured and IMRT is more widely used as standard treatment in clinics.
